# Antimicrobial Resistance or Delayed Appropriate Therapy—Does One Influence Outcomes More Than the Other Among Patients With Serious Infections Due to Carbapenem-Resistant Versus Carbapenem-Susceptible Enterobacteriaceae?

**DOI:** 10.1093/ofid/ofz194

**Published:** 2019-04-23

**Authors:** Thomas P Lodise, Ariel Berger, Arman Altincatal, Rosa Wang, Tarun Bhagnani, Patrick Gillard, Nicole G Bonine

**Affiliations:** 1 Pharmacy Practice, Albany College of Pharmacy and Health Sciences, Albany, New York; 2 Real-World Evidence, Evidera, Waltham, Massachusetts; 3 Global Health Outcomes Strategy & Research, Allergan plc, Madison, New Jersey

**Keywords:** antibacterial drug resistance, antibiotic resistance, carbapenems, cost of illness, Enterobacteriaceae

## Abstract

**Background:**

The relative contribution of antimicrobial resistance versus delayed appropriate treatment to the clinical and economic burden of Enterobacteriaceae infections is not well understood.

**Methods:**

Using a large US hospital database, we identified all admissions between July 2011 and September 2014 with evidence of serious Enterobacteriaceae infection. The “index date” was the earliest date on which a culture positive for Enterobacteriaceae was drawn. Infections were classified as carbapenem-resistant (CRE) or carbapenem-susceptible (CSE). Receipt of antimicrobials with activity against all index pathogens on the index date or ≤2 days thereafter was deemed as “timely”; all other instances were “delayed.” Associations between CRE status and delayed appropriate therapy on outcomes were estimated using inverse probability weighting and multivariate regression models (ie, logistic model for discharge destination and composite mortality [in-hospital death or discharge to hospice] or generalized linear model for duration of antibiotic therapy, hospital length of stay [LOS], and costs).

**Results:**

A total of 50 069 patients met selection criteria; 514 patients (1.0%) had CRE. Overall, 67.5% of CSE patients (vs 44.6%, CRE) received timely appropriate therapy (*P* < .01). Irrespective of CRE status, patients who received delayed appropriate therapy had longer durations of antibiotic therapy and LOS, higher costs, lower likelihood of discharge to home, and greater likelihood of the composite mortality outcome (*P* for trend < .01).

**Conclusions:**

Delayed appropriate therapy is a more important driver of outcomes than CRE, although the 2 factors are somewhat synergistic. Better methods of early CRE identification may improve outcomes in this patient population.

## Teaser

Although resistance and delayed appropriate therapy both are associated with worse clinical and economic outcomes, delayed therapy appears to be the more important factor. As these 2 measures are correlated, earlier identification of CRE may improve outcomes.

Enterobacteriaceae (eg, *Enterobacter* sp., *Escherichia coli*, *Klebsiella pneumonia*e) are commonly implicated in serious infections among adult hospitalized patients and are associated with considerable morbidity and mortality [1–4]. Treatment of these pathogens has been complicated by the continued emergence of antibiotic-resistant strains, with carbapenem-resistant Enterobacteriaceae (CRE) being of greatest concern from a public health perspective [5]. Relative to infections due to carbapenem-susceptible Enterobacteriaceae, patients with CRE infections have extended lengths of stay (LOS) in hospital, higher mortality rates, and greater healthcare costs [6–8].

Patients with CRE infections often receive inappropriate or delayed therapy [8], and ample evidence exists highlighting the deleterious consequences of delayed therapy, for patients with serious infections due to Enterobacteriaceae [9–14]. Although worse outcomes are associated with both carbapenem resistance and delayed appropriate therapy among patients with serious infections due to Enterobacteriaceae, it is unclear whether the outcomes observed with delayed appropriate therapy are merely a surrogate for CRE or vice versa. To date, few have attempted to simultaneously ascertain the contribution of each factor on patient outcomes. The objective of this study was to assess the independent and combined impact of CRE and delayed appropriate therapy on clinical and economic outcomes among hospitalized US patients with serious infections due to Enterobacteriaceae.

## METHODS

### Data Source

To accomplish the study objectives, we used the Premier Hospital Database, which contains information for approximately 50 million admissions (~20% of US total) from >500 acute-care hospitals (teaching, non-teaching, urban, and rural) [15]. We limited attention to the approximately 150 hospitals for which microbiological data were available.

Detailed information for each admission also includes the following: (1) primary and secondary diagnoses (in International Classification of Diseases, Ninth Revision, Clinical Modification [ICD-9-CM] format); (2) medications dispensed (eg, days of medication dispensed); (3) primary and secondary procedures (in ICD-9-CM format); (4) LOS; (5) services rendered (eg, items, quantity, costs, charges for all departments [including pharmacy]); (6) discharge status and destination; and (7) third-party payer. The database is fully de-identified and compliant with the Health Insurance Portability and Accountability Act 1996.

### Sample Selection

The study population consisted of all patients with ≥1 admission to the hospital between July 1, 2011, and September 30, 2014, with evidence of an infection of interest (ie, complicated urinary tract infections [cUTI], complicated intra-abdominal infections [cIAI], bacteremia, and hospital-acquired pneumonia [HAP], including ventilator-associated pneumonia [VAP]). Selection algorithms for each infection type are given in Table 1. We designated the index date as the earliest date on which a microbiological culture positive for Enterobacteriaceae from a site consistent with the infection type was drawn. Patients were classified as carbapenem-resistant (CRE patients) or carbapenem-susceptible (CSE patients) based on corresponding susceptibility data. Carbapenem resistance was defined based on a finding of resistant or intermediate to ≥1 carbapenem (ie, doripenem, meropenem, imipenem, or ertapenem) [16–18]. We excluded patients who did not receive antibiotics ≤3 days following the index date (except those who died or were transferred to other hospitals within that period), and those who were transferred from other hospitals; died or were discharged alive on the index date; were <18 years old on the index date; had evidence of pregnancy or childbirth during the admission; had evidence of necrotizing fasciitis, gangrene, ecthyma gangrenosum, osteomyelitis, or other chronic infection; or had invalid or missing data for outcomes of interest.

**Table 1.** Criteria to Select cUTI, cIAI, Bacteremia, and HAP

Infection	Criteria
cUTI	1. Any (principal or secondary) discharge diagnosis (ICD-9-CM) of UTI and
	2. ≥1 positive cultures for Gram-negative bacteria from a site consistent with UTI (eg, urinary catheter)
	OR
	1. Any discharge diagnosis of other urinary tract complication and
	2. any catheter-related procedure or other diagnostic evidence or complication and
	3. ≥1 positive cultures for Gram-negative bacteria from a site consistent with UTI (eg, urinary catheter).
cIAI	1. Any discharge diagnosis of IAI and
	2. ≥1 procedures for laparotomy, laparoscopy, or percutaneous drainage and
	3. ≥1 positive cultures for Gram-negative bacteria from a site consistent with IAI (eg, gastric culture).
Bacteremia	1. Any discharge diagnosis of bacteremia (including sepsis) and
	2. ≥1 positive cultures for Gram-negative bacteria from a site consistent with bacteremia (eg, blood).
HAP	1. Any discharge diagnosis of pneumonia (including VAP) and
	2. ≥1 positive cultures for Gram-negative bacteria from a site consistent with pneumonia (eg, sputum) and
	3. index date ≥3 days following admission
	OR
	1. Any discharge diagnosis of pneumonia (excluding VAP) and
	2. ≥1 positive cultures for Gram-negative bacteria from a site consistent with pneumonia and
	3. index date ≤3 days following admission and evidence that source of pneumonia was nosocomial.

Abbreviations: cIAI, complicated intra-abdominal infection; cUTI, complicated urinary tract infection; HAP, hospital-acquired pneumonia; ICD-9-CM, *International Classification of Diseases*, Ninth Revision, Clinical Modification; UTI, urinary tract infection; VAP, ventilator-associated pneumonia.

### Measures

Patients’ demographic and clinical characteristics were based on available information during the qualifying admission and the prior 6-month period. Patient-level covariates included in the analysis were demographics (age, gender, race, and payer type); comorbidities (asthma, cerebrovascular disease, congestive heart failure, respiratory diseases, coronary heart disease, dementia, hemiplegia/paraplegia, immunocompromising conditions, liver disease, malnutrition, rheumatoid arthritis, peptic ulcer disease, peripheral vascular disease, rheumatic disease, renal failure, or diabetes); Charlson Comorbidity Index (CCI); infection-related measures (ie, source of infection [community-acquired, healthcare-associated, or other], infection type [cUTI, cIAI, BSI or HAP]); a resource intensity cost index (values >1 indicate patients with higher-than-average costs during the first 2 days in hospital); pre-index culture in-hospital measures (eg, use of antibiotics, LOS, evidence of use of corticosteroids, parenteral nutrition, or vasoactive medications before the index date); index culture drawn in the intensive care unit (ICU); all-cause hospitalizations in the prior month, 3 months, or 6 months; and infection-related hospitalizations in the prior month, 3 months, or 6 months. Hospital characteristics assessed included geographic region, geographic subregion, teaching facility, and number of hospital beds. A full list of demographics and clinical characteristics are listed in the Supplementary Data.

Appropriateness of antibiotic therapy was defined as receipt of antibiotic(s) with in vitro activity (defined as “susceptible” in the database) against all identified index pathogens (ie, Enterobacteriaceae plus any other pathogen identified on the index date). In instances where susceptibility could not be ascertained directly from the database (ie, a patient treated with ≥1 antibiotic for which no susceptibility data were generated) [16], algorithms to determine pathogen susceptibility were derived (Supplementary Data). For example, an ampicillin susceptible *E. coli* would be considered carbapenem susceptible if no carbapenem susceptibility data were reported. However, susceptibility to a beta-lactam would not be inferred from a non-β-lactam antibiotic. The earliest date on which all index pathogens were covered was deemed the date of initiation of appropriate therapy. We defined receipt of appropriate therapy on the index date or within the subsequent 2 days as “timely” and all subsequent days as “delayed.”

Outcomes of interest included duration of antibiotic therapy post-index culture, LOS post-index culture, total in-hospital costs to render care post-index culture, discharge destination, and the composite outcome of in-hospital death or discharge to hospice. The database contains day-of-stay information on costs of care. Accordingly, all costs associated with all services (eg, medical care, pharmacotherapy, or room and board) noted between the index date to discharge date were included in the analyses; costs accrued prior to the index date were excluded from consideration. We used the same methods for all cases in all hospitals across the years, 2011–2014, and did not assume costs were homogenous across patients.

### Statistical Analyses

Data were summarized using descriptive statistics. Means and standard deviation were used to describe continuous variables, and frequencies and percentages were used to describe categorical variables. In unadjusted comparative bivariate analyses, we used Student’s *t* tests and Wilcoxon rank-sum tests to determine the statistical significance of differences in continuous variables, as appropriate. Chi-square tests were used for categorical variables.

Propensity scores were generated for each patient with an infection caused by Enterobacteriaceae spp on the index date by means of a multivariate logistic regression model that estimated for each patient the probability (a single variable bound by 0 and 1) of having CRE (vs CSE) [19, 20]. Propensity scores were used as inverse probability-weighted (IPW) estimators in the multivariate analyses, which allowed for the inclusion of all available information while still balancing the comparative groups for differences that may confound analyses of interest. The IPW estimator was used as a weight in the regression model to obtain balanced distribution of characteristics between the groups of interest (CRE vs CSE). Additional details on propensity scores and the IPW estimators that were used are found in the Supplementary Data.

A number of regression models were created, each of which included the relationships of interest plus 1 additional covariate of interest (eg, CRE [vs CSE] plus receipt of delayed appropriate therapy [vs timely appropriate therapy] plus age). All such covariate-specific models were assessed to determine whether inclusion of the additional variable changed the estimate associated with the relationship of interest by >10%. Covariates that changed the estimate by <10% were excluded. All covariates that modified the delayed appropriate therapy-related estimate by >10% were assessed for colinearity. Those with an absolute value for their correlation coefficient >0.6 were deemed colinear, in which case only the variable with the larger impact on the delayed appropriate therapy-related estimate was selected for the final multivariate model. For each population and outcome of interest, the multivariate model consisted of the base model plus all covariates that met the 10% criterion and were deemed not to be colinear.

Multivariate linear regression modeling was used to examine adjusted mean duration of antibiotic therapy post-index culture, post-index culture LOS in hospital, and post-index culture costs of care. As part of our analyses, all continuous outcome measures were log-transformed prior to multivariate analysis and retransformed thereafter. LOS and duration of antibiotic therapy were examined using negative-binomial regression models with log-link functions, and total in-hospital costs were examined using generalized linear models fit to gamma distributions with log-link functions. Discharge destination (ie, home vs all other destinations) and the composite outcome of in-hospital death or discharge to hospice were evaluated using multivariate logistic regression models. In all instances, covariates added to each model were those that met the 10% criterion described above.

## RESULTS

### Study Population

**Figure 1.**
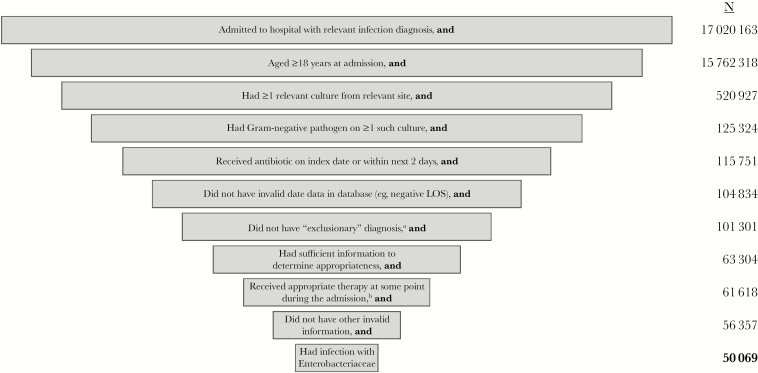
Full diagram of sample selection. LOS indicates length of stay. ^a^Including chronic infection (N = 1468), gangrene (N = 1468), necrotizing fasciitis (N = 186), osteomyelitis or related bone infection, or both (N = 1307), and pregnancy (N = 266). ^b^With exception of patients who expired relatively early in the course of admission (these patients were assumed to be “victims” of inappropriate empiric therapy and were therefore retained).

Among the 50 069 patients admitted during the study period who met all selection criteria (Figure 1), 52.6% had cUTI; 34.4%, bacteremia; 7.3%, HAP; and 5.8%, cIAI. A total of 514 patients (1.0% of the study sample) had infections caused by CRE, ranging from 2.9% (among patients with HAP) to 0.9% (among patients with either cUTI or bacteremia).

### CRE Versus CSE

Compared with CSE patients, those with CRE were more likely to be male (57.4% vs 43.7%); CRE patients also had higher mean CCI scores (Table 2; *P* < .01). CRE patients were about twice as likely to have received antibiotics during their qualifying admission prior to their index date and to be in the ICU on their index date. They also averaged 3.7 more days in hospital prior to the index date (all *P* < .01). CRE patients were more likely than CSE patients to have been hospitalized within 6 months of their qualifying admission.

**Table 2.** Demographic and Clinical Characteristics of Study Subjects, by CRE Status

	CRE Status^b^				Timing of Appropriate Therapy^d^			
Characteristic^a^	CRE (N = 514)	CSE (N = 49 555)	*P*-Value^c^	All Patients (N = 50 069)	Delay (N = 19 385)	Timely (N = 36 972)	*P*-Value^c^	All Patients (N = 56 357)
**Mean (SD) age, years**	66.0 (17.2)	65.7 (18.3)	.70	65.7 (18.3)	66.8 (16.6)	65.5 (18.9)	<.01	65.9 (18.1)
**Male**	295 (57.4)	21 677 (43.7)	<.01	21 972 (43.9)	9877 (51.0)	16 101 (43.5)	<.01	25 978 (46.1)
**Race**								
Caucasian	341 (66.3)	35 576 (71.8)	<.01	35 917 (71.7)	13 740 (70.9)	26 952 (72.9)	<.01	40 692 (72.2)
Black	119 (23.2)	8045 (16.2)		8164 (16.3)	3464 (17.9)	5669 (15.3)		9133 (16.2)
Other	54 (10.5)	5934 (12.0)		5988 (12.0)	2181 (11.3)	4351 (11.8)		6532 (11.6)
**Payer type**								
Commercial	77 (15.0)	8518 (17.2)	<.01	8595 (17.2)	3187 (16.4)	6201 (16.8)	<.01	9388 (16.7)
Medicaid	71 (13.8)	5779 (11.7)		5850 (11.7)	2209 (11.4)	4319 (11.7)		6528 (11.6)
Medicare	348 (67.7)	31 760 (64.1)		32 108 (64.1)	12 864 (66.4)	23 790 (64.3)		36 654 (65.0)
Other/unknown	18 (3.5)	3498 (7.1)		3516 (7.0)	1125 (5.8)	2662 (7.2)		3787 (6.7)
**Geographic Region**								
Northeast	182 (35.4)	11 003 (22.2)	<.01	11 185 (22.3)	4970 (25.6)	7689 (20.8)	<.01	12 659 (22.5)
Midwest	100 (19.5)	11 439 (23.1)		11 539 (23.0)	3974 (20.5)	9025 (24.4)		12 999 (23.1)
South	165 (32.1)	20 165 (40.7)		20 330 (40.6)	8005 (41.3)	15 002 (40.6)		23 007 (40.8)
West	67 (13.0)	6948 (14.0)		7015 (14.0)	2436 (12.6)	5256 (14.2)		7692 (13.6)
**Geographic sub-region**								
New England	15 (2.9)	2874 (5.8)	<.01	2889 (5.8)	1191 (6.1)	2055 (5.6)	<.01	3246 (5.8)
Middle Atlantic	167 (32.5)	8129 (16.4)		8296 (16.6)	3779 (19.5)	5634 (15.2)		9413 (16.7)
East North Central	87 (16.9)	6899 (13.9)		6986 (14.0)	2533 (13.1)	5406 (14.6)		7939 (14.1)
West North Central	13 (2.5)	4540 (9.2)		4553 (9.1)	1441 (7.4)	3619 (9.8)		5060 (9.0)
South Atlantic	101 (19.6)	14 076 (28.4)		14 177 (28.3)	5859 (30.2)	10 151 (27.5)		16 010 (28.4)
East South Central	30 (5.8)	2244 (4.5)		2274 (4.5)	909 (4.7)	1744 (4.7)		2653 (4.7)
West South Central	34 (6.6)	3845 (7.8)		3879 (7.7)	1237 (6.4)	3107 (8.4)		4344 (7.7)
Mountain	7 (1.4)	615 (1.2)		622 (1.2)	242 (1.2)	463 (1.3)		705 (1.3)
Pacific	60 (11.7)	6333 (12.8)		6393 (12.8)	2194 (11.3)	4793 (13.0)		6987 (12.4)
**Teaching facility**	269 (52.3)	20 867 (42.1)	<.01	21 136 (42.2)	9315 (48.1)	14 813 (40.1)	<.01	24 128 (42.8)
**Comorbidities**								
Asthma	26 (5.1)	3128 (6.3)	.24	3154 (6.3)	1178 (6.1)	2377 (6.4)	.1	3555 (6.3)
Cerebrovascular disease	2 (0.4)	92 (0.2)	.25	94 (0.2)	65 (0.3)	47 (0.1)	<.01	112 (0.2)
Congestive heart failure	131 (25.5)	9738 (19.7)	<.01	9869 (19.7)	4804 (24.8)	6788 (18.4)	<.01	11 592 (20.6)
Respiratory diseases (COPD + other chronic pulmonary disease)	300 (58.4)	20 306 (41.0)	<.01	20 606 (41.2)	10 565 (54.5)	13 896 (37.6)	<.01	24 461 (43.4)
Coronary heart disease (myocardial infarction + other)	62 (12.1)	4704 (9.5)	.05	4766 (9.5)	2202 (11.4)	3253 (8.8)	<.01	5455 (9.7)
Dementia	6 (1.2)	725 (1.5)	.58	731 (1.5)	304 (1.6)	531 (1.4)	.22	835 (1.5)
Hemiplegia/paraplegia	26 (5.1)	1509 (3.0)	<.01	1535 (3.1)	814 (4.2)	1072 (2.9)	<.01	1886 (3.3)
Immunocompromising conditions	157 (30.5)	11 386 (23.0)	<.01	11 543 (23.1)	5323 (27.5)	7926 (21.4)	<.01	13 249 (23.5)
HIV/AIDS	2 (0.4)	317 (0.6)	.78	319 (0.6)	139 (0.7)	236 (0.6)	.27	375 (0.7)
Cancer	90 (17.5)	6596 (13.3)	<.01	6686 (13.4)	3287 (17.0)	4516 (12.2)	<.01	7803 (13.8)
Other immunocompromising conditions	87 (16.9)	5900 (11.9)	<.01	5987 (12.0)	2674 (13.8)	4134 (11.2)	<.01	6808 (12.1)
Liver disease	27 (5.3)	2327 (4.7)	.55	2354 (4.7)	1014 (5.2)	1556 (4.2)	<.01	2570 (4.6)
Malnutrition	147 (28.6)	7563 (15.3)	<.01	7710 (15.4)	4555 (23.5)	4621 (12.5)	<.01	9176 (16.3)
Rheumatoid arthritis	10 (1.9)	1176 (2.4)	.53	1186 (2.4)	418 (2.2)	901 (2.4)	.04	1319 (2.3)
Peptic ulcer disease	9 (1.8)	429 (0.9)	.03	438 (0.9)	261 (1.3)	238 (0.6)	<.01	499 (0.9)
Peripheral vascular disease	36 (7.0)	3030 (6.1)	.4	3066 (6.1)	1424 (7.3)	2211 (6.0)	<.01	3635 (6.4)
Rheumatic disease	17 (3.3)	1681 (3.4)	.92	1698 (3.4)	641 (3.3)	1266 (3.4)	.46	1907 (3.4)
Renal failure (acute and chronic)	305 (59.3)	23 156 (46.7)	<.01	23 461 (46.9)	10 510 (54.2)	16 062 (43.4)	<.01	26 572 (47.1)
Diabetes	201 (39.1)	16 906 (34.1)	.02	17 107 (34.2)	6827 (35.2)	12 361 (33.4)	<.01	19 188 (34.0)
Diabetes, no chronic complications	151 (29.4)	13 154 (26.5)	.66	13 305 (26.6)	5236 (27.0)	9688 (26.2)	.04	14 924 (26.5)
Diabetes, with chronic complications	15 (2.9)	1292 (2.6)	.66	1307 (2.6)	611 (3.2)	855 (2.3)	<.01	1466 (2.6)
**Mean (SD) CCI**	3.6 (2.6)	3.0 (2.6)	<.01	3.0 (2.6)	3.5 (2.7)	2.8 (2.6)	<.01	3.1 (2.6)
**Source of infection**								
Community acquired	181 (35.2)	29 120 (58.8)	<.01	29 301 (58.5)	8898 (45.9)	22 423 (60.6)	<.01	31 321 (55.6)
Healthcare-associated	145 (28.2)	11 027 (22.3)	<.01	11, 172 (22.3)	4262 (22.0)	8388 (22.7)	.06	12 650 (22.4)
Nosocomial	188 (36.6)	9408 (19.0)	<.01	9596 (19.2)	6225 (32.1)	6161 (16.7)	<.01	12 386 (22.0)
**Type of infection**								
cUTI	26 096 (52.7)	227 (44.2)	<.01	26 323 (52.6)	7547 (38.9)	21 437 (58.0)	<.01	28 984 (51.4)
cIAI	2849 (5.8)	31 (6.0)	.77	2880 (5.7)	1576 (8.1))	1504 (4.1)	<.01	3080 (5.5)
Bloodstream infection	17 074 (34.4)	152 (29.6)	.02	17 226 (34.4)	7093 (36.6)	11 688 (31.6)	.6315	18 781 (33.3)
HAP	3536 (7.1)	104 (20.2)	<.01	3640 (7.3)	3169 (16.3)	2343 (6.3)	<.01	5512 (9.8)
**Resource intensity cost index**	0.9 (0.8)	0.7 (0.7)	<.01	0.7 (0.7)	1.2 (1.0)	0.9 (1.0)	<.01	1.0 (1.0)
**Any use of antibiotics before index date**	189 (36.8)	7682 (15.5)	<.01	7871 (15.7)	5542 (28.6)	4929 (13.3)	<.01	10 471 (18.6)
**Mean (SD) number of days in hospital before index date, d**	5.3 (10.6)	1.6 (5.4)	<.01	1.7 (5.5)	3.1 (9.9)	1.4 (4.7)	<.01	2.0 (7.0)
**Index culture drawn in ICU**	220 (42.8)	13 242 (26.7)	<.01	13 462 (26.9)	7156 (36.9)	8574 (23.2)	<.01	15 730 (27.9)
**Evidence of use on index day or day prior of**								
Corticosteroids	17 (3.3)	1330 (2.7)	.38	1347 (2.7)	764 (3.9)	845 (2.3)	<.01	1609 (2.9)
Parenteral nutrition	41 (8.0)	1656 (3.3)	<.01	1697 (3.4)	1256 (6.5)	731 (2.0)	<.01	1987 (3.5)
Vasoactive medications	110 (21.4)	6786 (13.7)	<.01	6896 (13.8)	4070 (21.0)	3953 (10.7)	<.01	8023 (14.2)
**Number of hospital beds**								
<100	2 (0.4)	965 (1.9)	<.01	967 (1.9)	258 (1.3)	809 (2.2)	<.01	1067 (1.9)
100–299	151 (29.4)	16 316 (32.9)		16 467 (32.9)	5700 (29.4)	12 602 (34.1)		18 302 (32.5)
300–499	185 (36.0)	17 426 (35.2)		17 611 (35.2)	6774 (34.9)	12 997 (35.2)		19 771 (35.1)
≥500	176 (34.2)	14 848 (30.0)		15 024 (30.0)	6653 (34.3)	10 564 (28.6)		17 217 (30.5)
**All-cause hospitalizations in prior**								
Month	176 (34.2)	12 772 (25.8)	<.01	12 948 (25.9)	5633 (29.1)	9512 (25.7)	<.01	15 145 (26.9)
3 months	272 (52.9)	20 665 (41.7)	<.01	20 937 (41.8)	8754 (45.2)	15 481 (41.9)	<.01	24 235 (43.0)
6 months	317 (61.7)	25 680 (51.8)	<.01	25 997 (51.9)	10 590 (54.6)	19 294 (52.2)	<.01	29 884 (53.0)
**Infection-related hospitalizations prior to index date**								
Month	121 (23.5)	6077 (12.3)	<.01	6198 (12.4)	2892 (14.9)	4624 (12.5)	<.01	7516 (13.3)
3 months	207 (40.3)	11 304 (22.8)	<.01	11 511 (23.0)	5172 (26.7)	8530 (23.1)	<.01	13 702 (24.3)
6 months	246 (47.9)	15 124 (30.5)	<.01	15 370 (30.7)	6693 (34.5)	11 378 (30.8)	<.01	18 071 (32.1)

Abbreviations: CCI, Charlson Comorbidity Index; cIAI, complicated intra-abdominal infection; COPD, chronic obstructive pulmonary disease; CRE, carbapenem-resistant Enterobacteriaceae; CSE, carbapenem-susceptible Enterobacteriaceae; HAP, hospital-acquired pneumonia; ICU, intensive care unit; SD, standard deviation.

^a^Unless otherwise noted, all variables are n (%).

^b^Patients that had evidence of Enterobacteriaceae.^c^*P* values obtained using *t* test for continuous variables, chi-square test for nominal categorical variables, and Wilcoxon rank sum for ordinal categorical variables.

^d^Patients that had evidence of Gram-negative pathogens.

CRE patients were more likely than CSE patients to be infected with *Klebsiella* sp. (59.5% vs 23.3%), *Enterobacter* sp. (23.0% vs 7.0%), or *Serratia* sp. (6.2% vs 2.7%); they also were less likely to have *Escherichia* sp. (18.9% vs 69.3%) (all *P* < .01) (Table 3).

**Table 3.** Frequency Distribution of Enterobacteriaceae, by CRE Status or Timing of Receipt of Appropriate Therapy

	CRE Status			Timing of Appropriate Therapy			
Pathogen	CRE (N = 514)	CSE (N = 49 555)	*P* Value^a^	Delayed (N = 16 414)	Timely (N = 33 655)	*P* Value^a^	All Patients (N = 50 069)
*Klebsiella* sp	59.5	23.3	<.01	28.0	21.7	<.01	23.7
*Citrobacter* sp	2.9	3.3	.63	3.8	3.1	<.01	3.3
*Enterobacter* sp	23.0	7.0	<.01	9.5	6.0	<.01	7.1
*Escherichia* sp	18.9	69.3	<.01	62.7	71.7	<.01	68.8
*Serratia* sp	6.2	2.7	<.01	4.0	2.2	<.01	2.8

Abbreviations: CRE, carbapenem-resistant Enterobacteriaceae; CSE, carbapenem-susceptible Enterobacteriaceae.

^a^ *P* values obtained using *t* test for continuous variables, chi-square test for nominal categorical variables, and Wilcoxon rank sum for ordinal categorical variables.

By multivariate-adjusted analysis, following index culture and relative to CSE patients, CRE patients averaged 1.0 additional days of antibiotic therapy, 0.8 additional days in hospital, and $4651 more in total in-hospital costs of care; they also were twice as likely to be discharged to hospice or die in the hospital (all *P* < .01) (Table 4).

**Table 4.** Multivariate-Adjusted Analyses of Infection-Related Outcomes: CRE vs CSE

Outcome^a^	CRE (N = 514)	CSE (N = 49 555)
Adjusted mean (95% CI)		
Duration of antibiotic therapy (d)^b^	8.5 (8.2 to 8.7)^c^	7.5 (7.5 to 7.5)
LOS (d)^b^	8.4 (8.2 to 8.7)^c^	7.6 (7.6 to 7.7)
In-hospital cost ($)^b^	19 816 (19 637 to 19 997)^c^	15 165 (15 031 to 15 300)
Adjusted OR (95% CI)^d^		
Discharged home	0.3 (0.3 to 0.3)^c^	
In-hospital death or discharged to hospice	2.2 (2.1 to 2.2)^c^	

Abbreviations: CRE, carbapenem-resistant Enterobacteriaceae; CSE, carbapenem-susceptible Enterobacteriaceae; LOS, length of stay; OR, odds ratio.

^a^Each outcome was adjusted for variables that were included in the inverse probability weighting: age, gender, race, payer type, geographic region, geographic subregion, teaching facility, comorbidities (ie, asthma, cerebrovascular disease, congestive heart failure, respiratory diseases, coronary heart disease, dementia, hemiplegia/paraplegia, immunocompromising conditions, liver disease, malnutrition, rheumatoid arthritis, peptic ulcer disease, peripheral vascular disease, rheumatic disease, renal failure, and diabetes with or without complications), Charlson Comorbidity Index score, source of infection, type of infection, resource intensity cost index, any use of antibiotic with coverage before index day, pre-index LOS, index culture drawn in the intensive care unit, any use of medications (corticosteroids, parenteral nutrition, and vasoactive) before index day, number of hospital beds, all-cause hospitalizations (in prior month, 3 months, or 6 months), and infection-related hospitalizations (in prior month, 3 months, or 6 months).

^b^Post-index culture.

^c^ *P* < .01.

^d^ The reference group was patients with CSE infections.

### Delayed Versus Timely Appropriate Therapy

Compared with patients who received timely appropriate therapy, those in whom therapy was delayed were more likely to be male (48.9% vs 41.5%); they also differed by race, payer type, and region (all *P* < .01) (Table 2). Patients who received delayed appropriate therapy were more likely than those who received timely appropriate therapy to have various comorbidities, nosocomial infection, and evidence of parenteral nutrition or vasoactive medications, or both, on the index date or the day prior (all *P* < .01). Patients who received delayed therapy were about 1.5 times as likely to be in the ICU on their index date and averaged around twice as many days in hospital prior to the index date (both *P* < .01).

Patients who received delayed appropriate therapy were more likely than those in whom therapy was deemed timely to have infections due to *Klebsiella* sp. (28.0% vs 21.7%), *Enterobacter* sp. (9.5% vs 6.0%), *Serratia* sp. (4.0% vs 2.2%), or *Citrobacter* sp. (3.8% vs 3.1%), and were less likely to have infections due to *Escherichia* sp. (62.7% vs 71.7%) (all *P* < .01) (Table 3).

### Stratified Analyses

**Figure 2.**
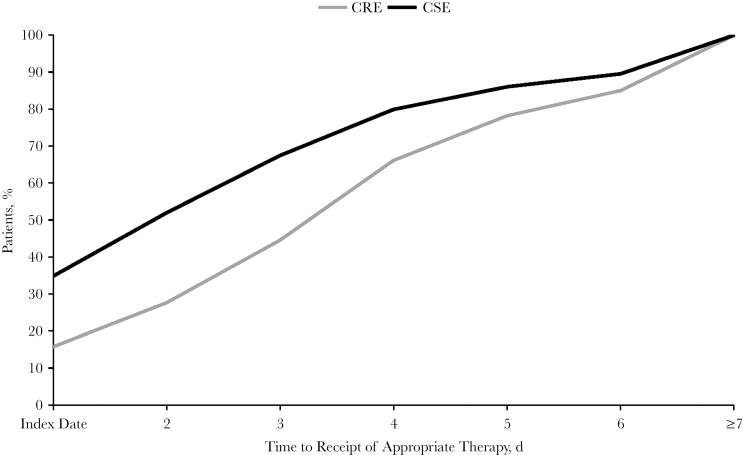
Time to receipt of appropriate therapy, by CRE status. CRE indicates carbapenem-resistant Enterobacteriaceae; CSE, carbapenem-susceptible Enterobacteriaceae.

Of CSE patients, 34.8%, 17.2%, and 15.5% received appropriate therapy on the index day (or “day 1”), day 2, or day 3, respectively; corresponding values for CRE patients were 15.8%, 11.9%, and 16.9%, respectively. Cumulative time to receipt of appropriate therapy is summarized in Figure 2. Approximately one-fifth of CRE patients received appropriate therapy on day 4.

After stratification, 55.4% (285 out of 514) of CRE patients were found to have received delayed appropriate therapy versus 32.5% (16 129 out of 49 555) of CSE patients (*P* < .01). Results of the multivariate-adjusted analyses are shown in Table 5. When both CRE and delayed appropriate therapy were included in the analyses, a gradient effect was observed across strata, with the worst outcomes experienced among the subgroup with CRE infection in whom appropriate therapy was delayed compared with the reference population (CSE infection who received timely appropriate therapy).

**Table 5.** Multivariate-Adjusted Analyses of Infection-Related Outcomes: CRE (vs CSE) and Receipt of Delayed Appropriate Therapy (vs Receipt of Timely Appropriate Therapy)

	Timely Appropriate Therapy		Delayed Appropriate Therapy	
Outcome^a^	CSE (N = 33 426)	CRE (N = 229)	CSE (N = 16 129)	CRE (N = 285)
Adjusted mean (95% CI)				
Duration of antibiotic therapy (d)^b,c^	5.0 (5.0 to 5.1)	5.4 (5.2 to 5.5)	8.3 (8.2 to 8.4)	8.9 (8.6 to 9.1)
LOS (d)^b,c^	5.0 (4.9 to 5.0)	5.1 (5.0 to 5.3)	8.5 (8.4 to 8.7)	8.8 (8.6 to 9.1)
In-hospital cost ($)^b,c^	9875 (9749 to 10 002)	11 539 (11 372 to 11 709)	21 828 (21 479 to 22 182)	25 506 (25 124 to 25 893)
Adjusted OR (95% CI)^d^				
Discharged home	Reference	0.4 (0.4 to 0.4)	0.4 (0.4 to 0.4)	0.2 (0.1 to 0.2)
In-hospital death or discharged to hospice	Reference	1.9 (1.9 to 2.0)	1.9 (1.8 to 2.0)	3.7 (3.5 to 3.9)

Abbreviations: CI, confidence interval; CRE, carbapenem-resistant Enterobacteriaceae; CSE, carbapenem-susceptible Enterobacteriaceae; IQR, interquartile ranges; LOS, length of stay; OR, odds ratio.

^a^Each outcome was adjusted for variables that were included in the inverse probability weighting: age, gender, race, payer type, geographic region, geographic subregion, teaching facility, comorbidities (ie, asthma, cerebrovascular disease, congestive heart failure, respiratory diseases, coronary heart disease, dementia, hemiplegia/paraplegia, immunocompromising conditions, liver disease, malnutrition, rheumatoid arthritis, peptic ulcer disease, peripheral vascular disease, rheumatic disease, renal failure, and diabetes with or without complications), Charlson Comorbidity Index score, source of infection, type of infection, resource intensity cost index, any use of antibiotic with coverage before index day, pre-index LOS, index culture drawn in the intensive care unit, any use of medications (corticosteroids, parenteral nutrition, and vasoactive) before index day, number of hospital beds, all-cause hospitalizations (in prior month, 3 months, or 6 months), and infection-related hospitalizations (in prior month, 3 months, or 6 months).

^b^Post-index culture.

^c^ *P* < .01.

^d^The reference group was patients with CSE infections who received timely appropriate therapy.

## DISCUSSION

This study sought to assess the degree to which pathogen susceptibility to carbapenems and delayed appropriate therapy, respectively, were associated with clinical and economic outcomes in hospitalized adults with serious infections due to Enterobacteriaceae. With few exceptions (eg, methicillin-resistant *Staphylococcus* aureus in skin and skin-structure infections), infection with a resistant pathogen tends to be highly correlated with receipt of delayed appropriate therapy [21, 22]. To ascertain their independent and combined impact, both IPW and stratified and multivariate analyses were performed to ensure clinical equipoise at baseline. Overall, our findings suggest that both CRE and receipt of delayed appropriate therapy negatively effect clinical and economic outcomes. However, delayed appropriate therapy was found to be a more important driver of outcomes relative to CRE status, although the 2 factors are somewhat synergistic. Interestingly, the clinical and economic outcomes of patients who received early versus delayed appropriate therapy were largely independent of CRE status. This indicates that it is not antibiotic resistance per se that effects clinical and economic outcomes, but whether appropriate antibiotic therapy is administered in a timely (or early) manner.

Our findings have important implications for clinical practice, as they suggest that the worse outcomes typically associated with Enterobacteriaceae infection, regardless of carbapenem susceptibility status, can potentially be mitigated by timely appropriate antimicrobial therapy. Our study therefore highlights the need for rapid diagnostics to shorten the lag time between clinical recognition of infection and downstream pathogen identification. Although rapid diagnostics accelerate time to pathogen reporting, current technologies are only able to identify a limited number of antibiotic-resistant Gram-negative pathogens. Therefore, decision-support system tools also are needed for the identification of patients at high risk for infections due to highly resistant pathogens such as CRE, the majority of whom likely also will be at high risk for delayed appropriate therapy. Such tools will be critically important for clinicians when selecting empirical treatment for patients, as culture results typically are not available within the first 48–72 hours of infection onset.

Several limitations of our study warrant discussion. First, our intent was to assess the independent and combined importance of antibiotic resistance and delayed treatment on observed outcomes. In reality, additional factors may be associated with the exposures and outcomes of interest (ie, our results may suffer from residual confounding), and caution is warranted in interpretation of our findings. Further research is needed to better understand the degree to which each outcome of interest is attributable to delayed appropriate therapy or CRE.

Second, as with all electronic health databases, there may be errors of omission or commission in coding. As our operational definitions were based on information within the database, study measures may be less accurate than those based on medical record review or data gathered prospectively.

Third, although we included a number of proxy measures for patients’ disease severity (eg, whether the index culture was taken while the patient was in the ICU, CCI score, resource intensity index), the database did not contain detailed clinical information to calculate acute disease severity measures like the acute physiology and chronic health examination (APACHE-II) or Pitt bacteremia score [23–26]. The effect of the inability to include an acute disease severity measure on the observed early versus delayed treatment results is unknown, but it is likely to be minimal given the number of proxies that were included and stratification of outcomes by CRE status, a known indicator of acute disease severity. Moreover, the database lacks information on healthcare utilization that occurred outside of Premier facilities, which likely render patients’ demographic and clinical characteristics incomplete.

Fourth, we defined CRE based on nonsusceptibility to carbapenems [27]. Although our definition had high specificity, its sensitivity is unknown. It is important to note that CLSI lowered the carbapenem susceptibility breakpoints during the study period, and it is possible that certain instances of CRE may have been classified as CSE. However, any resulting misclassification of patients should result in smaller differences between CRE and CSE patients and between patients in whom receipt of appropriate therapy was deemed timely versus delayed. Similarly, to the extent that we misclassified patients with asymptomatic bacteriuria or wound or drain colonization as having active (and complicated) infection, we believe that our results would tend to minimize the impact of timely appropriate therapy (ie, receipt of antibiotics in the absence of infection should have no impact on clinical and economic outcomes). Consequently, we believe that any such regression-to-the mean effect would render our findings somewhat conservative, and the deleterious clinical and economic consequences of delayed appropriate therapy may in fact be greater than those observed in our study.

Fifth, we determined the appropriateness of therapy based on in vitro susceptibility data contained in the database. In circumstances in which specific antibiotic-pathogen tests were unavailable, conservative algorithms were used to infer antibiotic susceptibility and appropriateness of therapy. Despite this, 37 997 Gram-negative infections were excluded due to lack of sufficient information to determine appropriateness. Of note, not all excluded episodes were of patients with infections due to Enterobacteriaceae, but rather reflected all patients with Gram-negative pathogens who were excluded due to lack of sufficient information to determine appropriateness. It is possible that exclusion of these patients could have possibly biased the results; nevertheless, the study sample (~50 000) was likely of sufficient power to answer the study questions.

Finally, our analyses focused attention on the most common Enterobacteriaceae. Although these patients represented the majority of those with CRE, exclusion of patients with relatively uncommon pathogens may limit the generalizability of our findings. Similarly, although our study reflects the experience of approximately 50 000 patients treated in approximately 150 hospitals across the US, the database is a convenience—and not a random—sample. Others have used the term appropriateness to include use of antibiotics only when necessary or administration of such agents at the proper dose and duration [28], both of which were beyond the scope of our study.

## CONCLUSIONS

In conclusion, CRE and delayed appropriate therapy both are associated with worse clinical outcomes and higher costs and charges among patients in US hospitals with serious infections due to Enterobacteriaceae. Although the impact of delayed appropriate therapy appears stronger than that of CRE, the effects of these 2 characteristics are synergistic. Given the association between these 2 factors, better methods of early identification of the causal pathogen(s) (particularly those hard to treat, such as CRE) should improve outcomes in this patient population.

## Supplementary Data

Supplementary materials are available at *Open Forum Infectious Diseases* online. Consisting of data provided by the authors to benefit the reader, the posted materials are not copyedited and are the sole responsibility of the authors, so questions or comments should be addressed to the corresponding author.

ofz194_suppl_supplementary_material
