# Supplementary material for: Antimicrobial Resistance or Delayed Appropriate Therapy—Does One Influence Outcomes More Than the Other Among Patients With Serious Infections Due to Carbapenem-Resistant Versus Carbapenem-Susceptible Enterobacteriaceae?
Source: Open Forum Infect Dis. 2019 Apr 23;6(6):ofz194. doi: 10.1093/ofid/ofz194 (PMC6546203; doi:10.1093/ofid/ofz194)

Antimicrobial Resistance or Delayed Appropriate Therapy—Does One Influence Outcomes More Than the Other among Patients with Serious Infections due to carbapenem resistant or carbapenem susceptible Enterobacteriaceae

Running Title: Resistance or Delay?

Author(s): Thomas Lodise, Ariel Berger, Arman Altincatal, Rosa Wang, Tarun Bhagnani, Patrick Gillard, PharmD, Nicole G. Bonine, PhD, MPH

Supplemental Materials

SAMPLE SELECTION

Briefly, the population consisted of all patients in the Premier inpatient research database with ≥1 admissions to hospital between July 1, 2011 and September 30, 2014 with evidence of a Gram-negative infection of interest (ie, HAP, BSI, cUTI, cIAI). The date of the earliest culture drawn from a relevant site (eg, urine for cUTI; sputum for HAP) positive for ≥1 Gram-negative bacteria of interest was deemed the “index date.”

The exclusion criteria were:

- patients transferred from other hospitals;
- died or were discharged alive on the index date;
- were aged <18 years;
- were pregnant/had evidence of childbirth (based on information from the admission) for relevant ICD-9-CM diagnosis codes;
- had evidence of infection with necrotizing fasciitis, gangrene, ecthyma gangrenosum, osteomyelitis, or other chronic infection for relevant ICD-9-CM diagnosis codes; these patients may be treated very differently than those with acute infections;
- had no evidence of receipt of antibiotics on the index date or within the 2-day subsequent period (ie, a 3-day period overall);
- did not have valid pathogen-antibiotic sensitivity test results; and/or
- could not have appropriateness of empiric therapy ascertained (eg, a patient for whom no susceptibility information specific to his/her empiric regimen was available and

for whom none of the “proxy” rules developed specifically for this project [eg, assume all fluoroquinolones have the same susceptibilities] applied).

COVARIATE MEASURES TO DETERMINE IPW

Demographic and Clinical Characteristics

These measures were used to characterize the study population and to potentially serve to ensure that the cohorts were at clinical equipoise as of index date. Patient characteristics included age, gender, payer type, comorbidities (eg, asthma, cerebrovascular disease, congestive heart disease, respiratory diseases, coronary heart disease, dementia, hemiplegia/paraplegia, immunocompromising conditions, liver disease, malnutrition, rheumatoid arthritis, peptic ulcer disease, peripheral vascular disease, rheumatic disease, renal failure, diabetes), composite comorbidity indices (ie, Charlson Comorbidity Index),[1] type of infection (eg, cUTI, cIAI, BSI, HAP), source of infection (ie, community-acquired, healthcare-associated, nosocomial), resource intensity index cost, any use of antibiotics prior to index date, mean LOS before index date, proxy for infection severity (index culture drawn in the ICU) any use of corticosteroids, parenteral nutrition or vasoactive medications), all-cause or infection-related hospitalizations in the prior month, 3 or 6 months. For any given patient, comorbidities were assessed based on the presence of at least one relevant diagnosis code (in ICD-9-CM format) in any diagnosis position (ie, primary *or* secondary) during the index admission.

Because the database only included month and year of admission, we assumed that all other (ie, non-infection-related) admissions that were identified during the month of the “qualifying” admission occurred prior to that admission. For example, a patient with an admission for

myocardial infarction (MI) and another admission during which a cUTI was identified would be assumed to have developed the cUTI subsequent to discharge for the MI.

Hospital characteristics included teaching facility (teaching vs non-teaching), geographic area (Northeast, Midwest, South, West), geographic division (New England, Middle Atlantic, East North Central, West North Central, South Atlantic, East South Central, West South Central, Mountain, Pacific), and number of beds. The difference between unweighted and weighted differences to demonstrate the degree to which IPW rendered the two patient groups balanced at baseline is shown in **Figure S1**.

Outcomes

Outcomes of interest during the qualifying admission include the following:

- Duration of antibiotic therapy, defined as beginning on the earliest date on or after the index date during which antibiotics were received, and ending on the last date of receipt of such therapy during the same "qualifying admission" or date of discharge (whichever occurred first), as shown below:

$$(AbxLAST - AbxFIRST) + 1;$$

where "AbxLAST" was the last date of antibiotic therapy during the admission and "AbxFIRST" was the earliest date of receipt of antibiotic therapy. Duration of antibiotic therapy was assessed for all patients in each relevant sample, irrespective of discharge status (ie, alive vs dead).

- LOS in hospital, defined as beginning on the index date and ending on the date of discharge, as shown below:

  $$(DischargeDATE - \ IndexDATE) + 1;$$

  where "DischargeDATE was the discharge date and "IndexDATE" was the index date.

- In-hospital mortality, defined based on discharge status (ie, alive vs dead) during the "qualifying admission", and was assessed for all patients in each relevant sample.
- Total costs of care, defined in a similar manner to all-cause costs, but was limited to the period spanning the index date to the date of discharge
-

  $$IndexAbxCOST + IndexOtherRxCOST + IndexMedCareCOST + IndexR\&BCOST$$

  where "IndexAbxCOST" was the cost of antibiotics from index date to date of discharge; "IndexOtherRxCOST", the cost of all other pharmacotherapy from index date to date of discharge; "IndexMedCareCOST", the cost of all other medical care from index date to date of discharge; and "IndexR&BCOST", the cost of room and board from index date to date of discharge. Costs of care were assessed for all patients in each relevant sample, irrespective of discharge status (ie, alive vs dead).

- Discharge destination, defined based on discharge destination (eg, home, long-term care facility, skilled nursing facility, hospice) during the "qualifying admission."

- In-hospital mortality or discharge to hospice, a composite measure based on discharge status and destination that was defined as *either* in-hospital mortality (as described above) *or* discharge to hospice (as described above).

STATISTICAL ANALYSIS

Descriptive statistics (means, standard deviations [SDs], medians, interquartile ranges [IQRs]) were used to describe continuous variables. Frequencies and percentages were used to describe categorical variables. In comparative analyses focused on type of resistant pathogen (ie, CRE vs non-CRE, CRP vs non-CRP, MDRP vs non-MDRP), the demographic and clinical characteristics of patients with resistant infections were found to differ significantly from those of patients with susceptible infections.

Propensity scores were generated for each patient with Enterobacteriaeae infection on the index date for whom carbapenem-resistance status could be ascertained by means of a multivariate logistic regression model that estimated for each patient the probability (a single variable bound by 0 and 1) of having CRE (vs. not). Covariates that were included in the models were described above. The general form of the model used to derive this probability, which is the propensity score, is shown below.

$$\log [p/(1-p)] = \beta_0 + \beta_1 * X_1 + \beta_2 * X_2 + \beta_N * X_N$$

Where p was the probability of being in one of the two groups of interest (eg, CRE); β_0, the intercept; β_1, the coefficient associated with the first model covariate; X_1, the first model covariate (these are typically expressed dichotomously as either 1 [ie, the covariate is present] or 0 [the covariate is absent]); β_2, the coefficient associated with the second model covariate; X_2,

the second model covariate; β_N, the coefficient associated with the "nth" model covariate; and X_N, the "nth" model covariate.

IPW estimators described above were used as weights in the regression models to obtain balanced distributions of characteristics between the groups of interest (eg, CRE vs non-CRE) [2]. The method by which these weights were used is illustrated below:

$$\sum_{i=1}^{N} w_i((y_i - (\beta_0 + \beta_1 * x_1 + \beta_2 * x_2 + \beta_N * x_N))^2$$

Where N is the total number of patients; y_i, the observed outcome in patient "i"; and w_i, the weight for patient "i" (of N) (all other variables are as described above).

References

1. McGregor JC, Perencevich EN, Furuno JP, et al. Comorbidity risk-adjustment measures were developed and validated for studies of antibiotic-resistant infections. *J Clin Epidemiol*. 2006;59:1266-1273.
2. Curtis LH, Hammill BG, Eisenstein EL, Kramer JM, Anstrom KJ. Using inverse probability-weighted estimators in comparative effectiveness analyses with observational databases. *Med Care*. 2007;45:S103-107.

Figure S1. Comparison of unweighted and weighted standardized differences: delayed vs timely appropriate therapy among patients with resistant infections

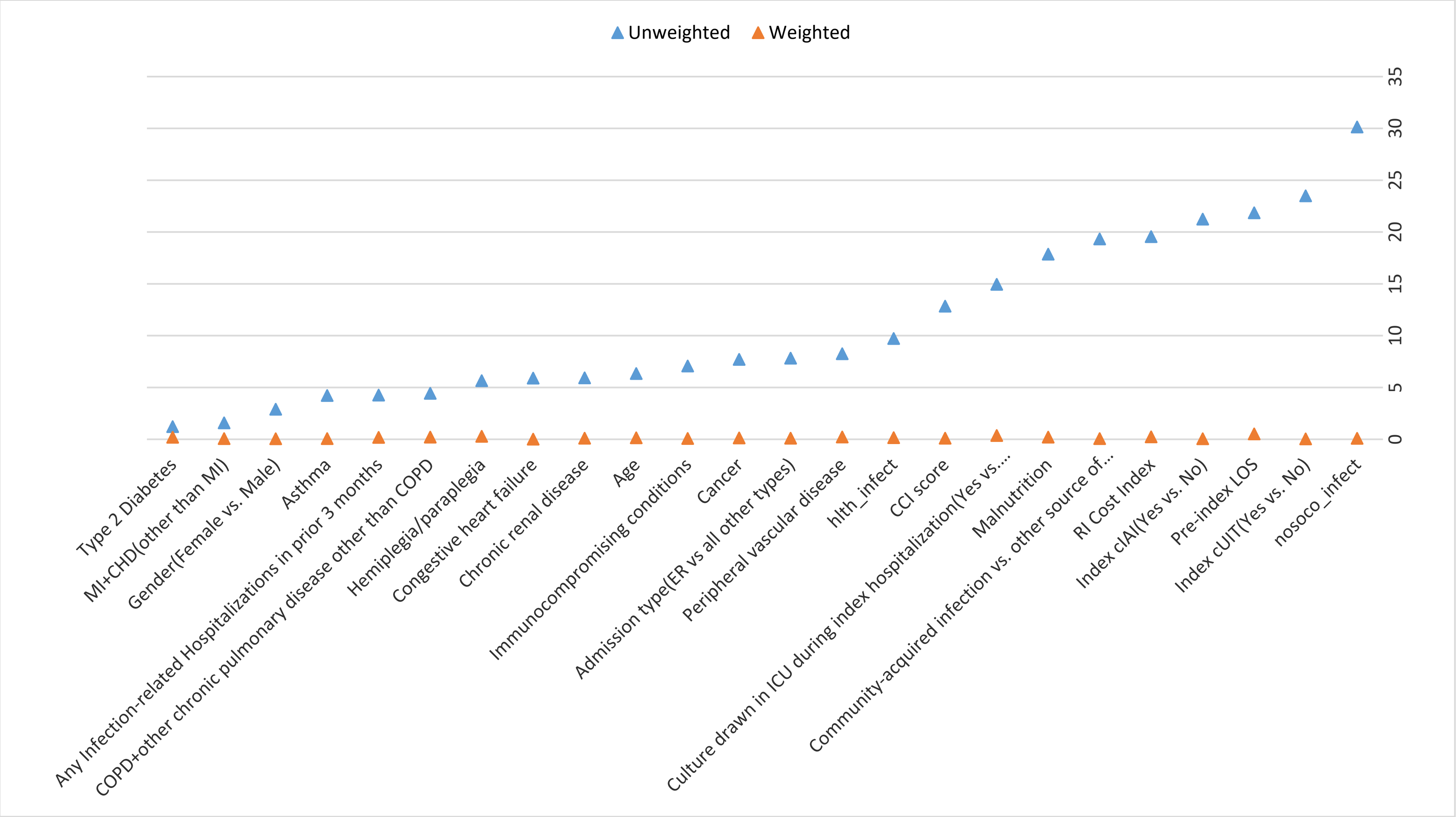

Supplement: ofz194_suppl_supplementary_material [file ofz194_suppl_supplementary_material.pdf]
